# Travellers as sentinels of chikungunya epidemics: a family cluster among Finnish travellers to Koh Lanta, Thailand, January 2019

**DOI:** 10.2807/1560-7917.ES.2019.24.11.1900162

**Published:** 2019-03-14

**Authors:** Anu Kantele

**Affiliations:** 1 Inflammation Center, Department of Infectious Diseases, Helsinki University Hospital and University of Helsinki, Helsinki, Finland

**Keywords:** chikungunya, cluster, Thailand, outbreak, travel, traveller

## Abstract

In January 2019, five of 11 travellers to Koh Lanta, Thailand, contracted chikungunya, symptoms starting 4 days after presumed transmission. Four cases were hospitalised, one child treated in intensive care; 6 weeks after disease onset, all three adults have persistent arthralgias/arthritis, incapacitating for two. Together with a recent report of eight chikungunya cases among travellers to various destinations in Thailand, the high attack rate in our cluster points to an ongoing outbreak in the country.

Recently, eight chikungunya cases were reported from various European countries and Israel, all returning from Thailand between November 2018 and February 2019 [1]. Here, we report a cluster of five chikungunya cases among 11 family members visiting Koh Lanta, an island district in Krabi Province, in the south of Thailand, in January 2019. Three of the eight cases in the earlier report had visited the same destination as this family [1].

## Case description

A vacation was taken by a group comprising 11 relatives in four family units: family one (adult family members 1–2), family two (adult family members 3–4 and children family members 5–6), family three (family members 7–8); family four (family members 9–11), resided at the same hotel in a popular tourist destination Koh Lanta, Thailand from mid-January 2019, having meals together. The ages of the family members ranged from 9 to 70 years, nine were adults, five were male. Their holiday trips were planned to last 1 or 2 weeks; family 4 returned to Finland after 1 week as scheduled.

On day 9 of their stay, family 2 (members 3–6) spent the evening on the beach practicing sports, while families 1 and 3 only visited the beach briefly. The following day, members of the local population mentioned that there had been an unusually high number of mosquitoes, a ‘mosquito invasion’, the evening before. Family members 3–6 had applied mosquito repellents that morning and reported only a few bites, while family member 1 had not used any repellent and reported 60–70 mosquito bites all over his legs. Family members 3–6 and family member 1 all fell ill 4–6 days later (days 23–25 of stay in Thailand) with a systemic febrile disease and arthralgias. A description of the cases (in the order of symptom onset) can be seen in Table 1.

**Table 1:** Cluster of five laboratory-confirmed chikungunya cases in family cluster in order of symptom onset, Koh Lanta, January 2019

Sex/age group (years)	Underlying diseases	Clinical symptoms	Outcome	Information available from hospital in Thailand	CHIKV diagnostic in Finland (day after symptom onset)	Other laboratory tests in Finland (day after symptom onset)
Male/5–9 (family member 5)	Diabetes mellitus type I	Fever, rash, arthralgia in wrists, elbows and back, diarrhoea	Return had to be delayed by one week; hospitalised in Thailand and in Finland; persistent arthralgia for 20 days **Evaluation on 1 March (day 35)**: Recovered fairly quickly after return to Finland, only minor arthralgias left	RDT for dengue positive, CHIKV antibodies positive; final diagnosis coinfection of dengue fever and chikungunya	Anti-CHIKV - IgM-positive; - IgG > 2,560 by ELISA (day 11)	Hb 103; leuc 8.2; tromb 541; eos 0.17; CRP 51; LD 266; blood culture negative; PCR for bacterial stool pathogens: enteroaggregative *E. coli*; stool PCR for protozoa negative; malaria tick and thin blood smears negative; influenza A and B by RDT negative; NS1 antigen and dengue virus antibodies negative (day 11)
Female/10–14 (family member 6)	Pollen allergy	Fever, poor condition, suspicion of septic shock, low blood pressure, rash, arthralgia in knees, heels and toes	Return had to be delayed by one week; intensive care in Thailand, treatment with intravenous ceftriaxone; hospitalised in Finland; persistent arthralgia for 28 days **Evaluation on 1 March (day 35):** Clinical picture initially more severe than for the others. Recovered fairly quickly after return to Finland, only minor arthralgias left.	RDT for dengue weak positive (day 2 of symptom onset), CHIKV antibodies positive; final diagnosis dengue fever and chikungunya	Anti-CHIKV - IgM–positive - IgG > 2,560 by ELISA (day 11)	Hb 133; leuc 4.2; tromb 463; eos 0.08; CRP < 3; ALAT 43; LD 326; blood culture negative; PCR for bacterial stool pathogens: negative; stool PCR for protozoa: negative; NS1 antigen and dengue virus antibodies negative; MDR colonisation negative for MRSA, ESBL-PE, CPE, VRE, multiresistant *Acinetobacter*, multiresistant *Pseudomonas* (day 11)
Male/70–74 (family member 1)	Diabetes mellitus II, hypertension, cardiovascular disease	Fever, arthralgia, polyarthritis, tenosynovitis, itching of skin (no rash)	Hospitalised in Thailand; persistent arthralgia in fingers, wrists and neck, clinical arthritis and tenosynovitis in right foot. Substantial walking difficulties, impaired capability to undertake daily activities. **Evaluation on 1 March (day 35):** Due to rivaroxaban medication, non-steroidal anti-inflammatory agents contraindicated. Received a one-week course of steroids Disabling arthralgias and arthritis continue; Visited today a rheumatologist and was given intra-articular steroids due to polyarthritis, treatment ongoing.	Unknown	Anti-CHIKV - IgM positive - IgG > 1,280 by ELISA (day 25)	Hb 148; leuc 8.2; tromb 272; eos 0.08; CRP < 3; ALAT 73; stool culture for Salmonella, Shigella, Campylobacter, Yersinia: negative; NS1 antigen and dengue virus antibodies negative (day 25)
Female/45–49 (family member 3)	Asthma bronchiale	Fever, arthralgia/pain in ankles, heels, achilles tendons, fingers, wrists; diarrhoea; rash	Return home had to be delayed by one week, persistent disabling arthralgia, walking difficulties. **Evaluation on 1 March (day 34):** Started on day 13 after symptom onset with etoricoxib low dose (30 mg), gradually increasing to 90 mg, then rash; now receiving ibuprofen, walking difficulties, will be referred to a rheumatologist unless starts to improve soon.	no visits to healthcare	Anti-CHIKV - IgM positive - IgG > 2,560 by ELISA (day 10)	Hb 148; leuc 4.6, part of lymphocytes reactive, a few plasma cells; tromb 286; eos 0.05; CRP 51; ALAT 39; ASAT 18; blood culture negative; stool PCR for bacteria: enteropathogenic *E. coli*; NS1 antigen negative; very low levels of dengue virus antibodies (day 10)
Male/45–49 (family member 4)	None	Fever; arthralgia, strong pain in heels, causing inability to walk, pain also in left knee, wrists, ankles; itching of skin (no rash)	Hospitalised in Thailand until day 4 after onset of symptoms; Return had to be delayed by one week; persistent arthralgia. **Evaluation on 1 March (day 33):** Started on day 20 after symptom onset ibuprofen; arthralgias continue but are improving and patient manages with current medication.	Dengue antigen and antibody, malaria thick and thin blood smears, influenza antigen all negative, CHIKV antibodies positive; final diagnosis chikungunya	Anti-CHIKV - IgM positive - IgG > 2,560 by ELISA (day 26)	Hb 157; leuc 8.6; tromb 415; eos 0.14; CRP < 3; ALAT 26; NS1 antigen and dengue virus antibodies by EIA negative (day 26)

ALAT: alanine aminotransferase; CRP: C-reactive protein; CHIKV: chikungunya virus; SPE: carbapenemase-producing *Enterobacteriaceae*; CRP: C-reactive protein; EIA: enzyme-immuno assay; eos: eosinophils; ESBL-PE: extended-spetrum beta-lactamase-producing *Enterobacteriaceae*; Hb: haemoglobin; LD: lactate dehydrogenase; Leuc: leucocyte; MRSA: methicillin resistant *Staphylococcus aureus*; NS1: non-structural protein 1; PCR: polymerase chain reaction; RDT: rapid diagnostic test; tromb: trombocytes; VRE: vancomycin-resistant *Enterococcus faecalis.*

Normal values: CRP (< 3 mg/l); eos (0.03-0-44 E9/l); Hb (117-156 g/l); LD (115-235 U/l); leuc (3.4-8.2 E9/l); tromb (150-360 E9/l).

Family 2 was scheduled to return home after a stay of 14 days, but due to the severity of disease in the children (family members 5 and 6), the flight was postponed by 1 week. On return to Finland, the children were further hospitalised. On 1 March (38 days after presumed transmission), the children are back at school with minor arthralgias remaining. Family member 1 continues to have severe polyarthralgia and arthritis, but cannot use nonsteroidal anti-inflammatory drugs (NSAIDs) due to other medications, and has therefore been referred to a rheumatologist. Family member 3 has asthma as underlying disease; while her condition improved with a low dose etoricoxib (30 mg OD), higher doses resulted in an allergic reaction. Referral to a rheumatologist is planned if further clinical improvement under ibuprofen therapy (600 mg TD) is not observed. Family member 4 has arthralgia, but he manages on ibuprofen medication.

## Discussion

Febrile systemic diseases in Thailand are not as common as other travel-related health problems like diarrhoea [2,3], but often are more severe. Febrile systemic diseases include a large variety of mosquito-borne infections such as malaria, lymphatic filariasis and viral infections caused by Japanese encephalitis, zika, dengue and chikungunya viruses [4,5]. Prevention of these diseases relies heavily on mosquito avoidance, as vaccines are currently only available for Japanese encephalitis [5]. Chikungunya virus (CHIKV) is transmitted, like dengue and zika viruses, by *Aedes aegypti* and *Aedes albopictus* mosquitoes [4,5] and has an incubation period of 3–7 days, symptoms beginning with an abrupt onset of fever followed by a predominating symptom of severe, sometimes disabling, polyarthralgia [6]. Some patients also develop a rash [6].

High attack rates of chikungunya pose a risk for tourists in areas where there is local transmission. Travellers can act as sentinels of outbreaks in a region. The cluster we present here accords with the findings by Javelle et al. [1] concerning Geosentinel sites describing a peak in chikungunya cases among visitors to Thailand. Of particular interest in our cluster is the high attack rate – with five of 11 travellers contracting chikungunya, all among the eight who stayed for longer than 1 week.

If, on return to their home countries, travellers are still viraemic and *Aedes* vectors prevail in their home country, there could be a risk of disease spread. *Ae. albopictus* appears to be the main vector for most of the European Union and European Economic Area, with Madeira as an exception since it also has an established *Ae. aegypti* population [7]. *Aedes* mosquitoes are only found in certain parts of central and southern Europe and the transmission period depends on temperature, usually not beginning before late spring [8].

In our cluster, four of the five cases had to be hospitalised in Thailand and one of the children required treatment in an intensive care unit. Severe manifestations and even deaths are seen in outbreaks, mostly among the elderly or those with underlying diseases [6]. Indeed, in our cluster, the two children had the most severe clinical picture in the acute phase and recovered fairly rapidly, whereas the symptoms persist longer among the adults: now, 6 weeks after the presumed transmission, the two children are nearly asymptomatic, while the three adults still have arthalgias/arthritis, for two of them incapacitating.

In one adult case, an underlying disease prevents the use of NSAIDs for post-acute chikungunya. Antirheumatic medications such as methotrexate and hydroxychloroquine are recommended alternatives, if the NSAIDs cannot be used [9]; while corticosteroids should not be taken in the acute phase, they may be needed in the chronic stage. Despite treatment, however, severe joint problems may continue for months or even years [6].

While travellers may act as sentinels of outbreaks in a destination, the peak observed in chikungunya cases in Thailand [1] calls for enhanced prevention also among forthcoming travellers. There is no vaccine available against chikungunya, and mosquito avoidance remains the sole preventive approach. Indeed, it also protects against dengue and zika viruses transmitted by the same mosquito species; even co-infections have been described [10,11]. Many travellers use mosquito repellents especially at dusk and dawn, the time malaria-transmitting *Anopheles* mosquitoes bite. However, since the *Aedes* mosquitoes mostly bite in the daytime [4,8], to shield themselves against chikungunya, dengue and zika viruses, travellers should also be advised to apply repellents during the day.

Thailand is a popular tourist destination in the world: in 2017 there were over 35 million visitors, 6.5 million arriving from Europe [12]. Many travellers to Thailand do not seek pre-travel advice [13] and it is thus a challenge reach them. Travel agencies, insurance companies and other actors outside the medical community should be approached as additional channels to reach out to tourists and deliver relevant information.
